# Epigenetic control and genomic imprinting dynamics of the Dlk1-Dio3 domain

**DOI:** 10.3389/fcell.2023.1328806

**Published:** 2023-12-12

**Authors:** Ariella Weinberg-Shukron, Neil A. Youngson, Anne C. Ferguson-Smith, Carol A. Edwards

**Affiliations:** ^1^ Department of Genetics, University of Cambridge, Cambridge, United Kingdom; ^2^ School of BioSciences, The University of Melbourne, Parkville, VIC, Australia

**Keywords:** Dlk1-Dio3 domain, genomic imprinting, CTCF, chromatin architecture, DNA methylation, long non-coding RNA

## Abstract

Genomic imprinting is an epigenetic process whereby genes are monoallelically expressed in a parent-of-origin-specific manner. Imprinted genes are frequently found clustered in the genome, likely illustrating their need for both shared regulatory control and functional inter-dependence. The *Dlk1-Dio3* domain is one of the largest imprinted clusters. Genes in this region are involved in development, behavior, and postnatal metabolism: failure to correctly regulate the domain leads to Kagami–Ogata or Temple syndromes in humans. The region contains many of the hallmarks of other imprinted domains, such as long non-coding RNAs and parental origin-specific CTCF binding. Recent studies have shown that the Dlk1-Dio3 domain is exquisitely regulated *via* a bipartite imprinting control region (ICR) which functions differently on the two parental chromosomes to establish monoallelic expression. Furthermore, the *Dlk1* gene displays a selective absence of imprinting in the neurogenic niche, illustrating the need for precise dosage modulation of this domain in different tissues. Here, we discuss the following: how differential epigenetic marks laid down in the gametes cause a cascade of events that leads to imprinting in the region, how this mechanism is selectively switched off in the neurogenic niche, and why studying this imprinted region has added a layer of sophistication to how we think about the hierarchical epigenetic control of genome function.

## Introduction

### Genomic imprinting in mammals

Genome function is regulated temporally and tissue specifically through the orchestrated interplay of regulatory factors, genomic features, and epigenetic states. Epigenetic modifications are dynamic during development and across the cell cycle. A hierarchy of successive epigenetic states, including DNA methylation, ensures the creation of healthy individuals. In mammals, extensive epigenetic reprogramming events occur during germ cell development, fertilization, and early embryogenesis (Smith and Meissner, 2013). Although DNA methylation is essential for normal mammalian development, there appear to be multiple ways in which it can regulate and maintain cell fate and function, which remain incompletely understood. Although intensively studied, the association between DNA methylation and transcription is often correlative, with little experimental evidence to support causal relationships.

One major process in which predictive and causal relationships between DNA methylation and gene expression is more comprehensively understood is that of mammalian genomic imprinting (Bartolomei and Ferguson-Smith, 2011; Ferguson-Smith, 2011). Genomic imprinting is an epigenetically regulated process causing genes to be expressed from one chromosome homolog according to the parent-of-origin. Imprinting is highly conserved in eutherian mammals, and mouse studies have provided insights into the repertoire of developmental and physiological pathways regulated by imprinted genes (Ferguson-Smith and Bourc’his, 2018; Cleaton et al., 2014). Failure to correctly establish and maintain imprints is associated with developmental syndromes, including growth abnormalities, neurological and metabolic disorders, and numerous forms of cancer (Uribe-Lewis et al., 2011; Ishida and Moore, 2013). Imprinted genes are also highly expressed in the developing and adult brain, and are implicated in numerous brain functions, including behavior (Keverne, 1997; Liu et al., 2010; Furutachi et al., 2013; Tsan et al., 2016). Several syndromes that result from dysregulation of imprinted loci involve brain dysfunction, such as Prader–Willi syndrome (PWS) (Aman et al., 2018), Angelman syndrome (Nicholls et al., 1998), Turner syndrome (Bondy, 2006; Lepage et al., 2013), autism (Flashner et al., 2013), bipolar depression (Pinto et al., 2011), and schizophrenia (Ingason et al., 2011; Isles et al., 2016).

Genomic imprinting in laboratory mice is a tractable model for studying epigenetic regulation as the two parentally inherited, genetically identical genomic regions within the same nucleus express a different repertoire of genes in a parental-origin-specific manner. Imprinting is established in the germ cells through the differential deposition of DNA methylation in the two parental germlines (Smallwood et al., 2011). Differential DNA methylation marks (DMRs) at imprinting control regions (ICRs) are maintained in the post-fertilization period and protected from the global methylation erasure in the early embryo by KRAB zinc finger proteins ZFP57 and ZFP445 in order to maintain the epigenetic memory of parental origin (Takahashi et al., 2019). However, the dynamic hierarchy of events initiated by the ICRs that leads to the long-range domain-wide temporal and tissue-specific behavior of imprinted genes is not fully understood.

Studies assessing global as well as locus-specific alterations to ICRs have emphasized that loss of imprinting results in reciprocal effects on imprinted genes with the biallelic expression of some genes within the cluster and biallelic repression at others (Tucker et al., 1996; Li et al., 2008; Demars and Gicquel, 2012; Azzi et al., 2014; Takahashi et al., 2019). Phenotypically, perturbations to individual imprinted genes exert effects in numerous developmental and physiological pathways (Kalish et al., 2014; Tucci et al., 2019). Together, this has led to the prevailing notion in the field that at least some imprinted genes are dosage-sensitive. Deletions or insertions of the genes themselves, and aberrations that disrupt the pattern of imprinted gene expression, like mutations in the ICR or uniparental disomy (UPD), contribute to tumor progression and disease. In addition, the balance between maternally and paternally inherited genes can modulate phenotypes. For instance, PWS patients with maternal UPD or with ICR deletions have increased maternal expression along with a loss of paternal gene expression. These individuals are far more associated with psychotic illnesses than PWS patients with individual paternal gene deletion genotypes (Nicholls et al., 1998; Tucci et al., 2019). Yet, because the intricate epigenetic control at imprinted clusters controls the parent-specific expression of multiple genes, it is difficult to assign the relative contribution of the individual gene dosage to the resulting physiological phenotypes. Utilizing a systemic set of mutants at a single imprinted domain allows us to dissect the relationship between allelic expression, dosage, epigenetic control, and phenotypical outcomes.

## Genomic imprinting at the Dlk1-Dio3 domain

One of the largest imprinted clusters in mammals is the 1.2 Mb Dlk1-Dio3 domain. This region is conserved between mice and humans, and is one of the major developmentally regulated mammalian imprinted domains. Failure to correctly imprint genes in this cluster in humans leads to Temple or Kagami–Ogata syndromes, both of which exhibit neurological, developmental, and behavioral impairments (Ioannides et al., 2014; Kagami et al., 2015). In mice, both maternal and paternal UPDs containing this domain lead to prenatal lethality, further illustrating the developmental importance of the correct dosage of genes in the region.

**FIGURE 1:**
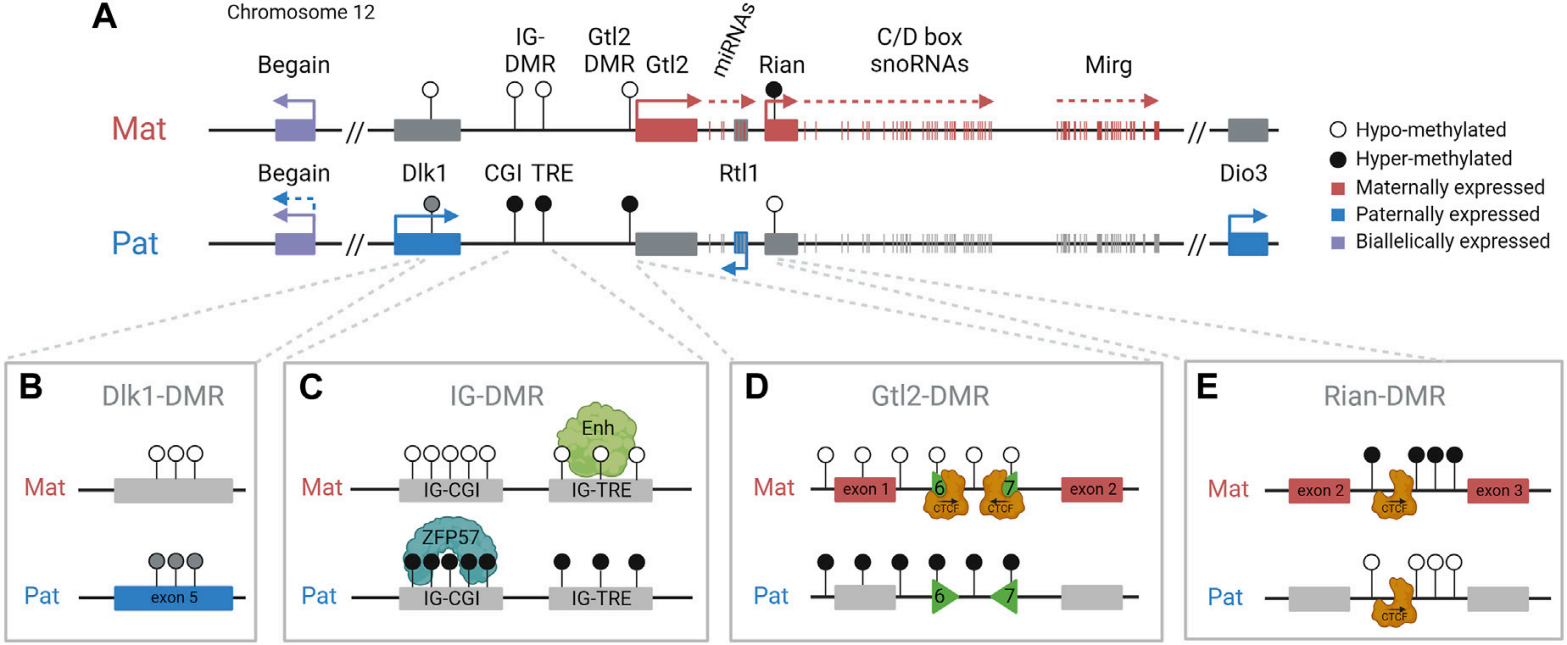
*Dlk1-Dio3* imprinted domain and key regulatory features. **(A)**. The paternally inherited chromosome expresses *Dlk1*, *Rtl1*, *Dio3*, and one isoform of *Begain*. The maternally inherited chromosome expresses *Gtl2/Meg3*, *antiRtl1*, and arrays of snoRNAs and miRNAs. The IG-DMR is methylated in sperm, is unmethylated in oocytes, and is the imprinting control region for the domain. DMRs are indicated by circles: black (methylated) and white (unmethylated). **(B)**. The somatic Dlk1-DMR, in the last exon of the *Dlk1* gene, is differentially methylated with partial methylation on the paternal chromosome. **(C)**. The IG-DMR contains a CpG island (CGI) that binds ZFP57 on the methylated paternal copy and a transcriptional regulatory element (TRE) that has an enhancer-like function on the unmethylated maternal copy. **(D)**. The Gtl2-DMR contains two differentially methylated CTCF binding sites (CTCF6 and 7) binding CTCF only on the unmethylated maternal chromosome. **(E)**. The Rian-DMR, in the second intron of the *Rian* gene, is methylated in the reverse pattern as the IG-DMR and Gtl2-DMR, methylated on the maternal chromosome, and hypomethylated on the paternal chromosome.

Four protein-coding genes, *Dlk1*, *Rtl1*, *Dio3*, and one isoform of *Begain* (located upstream of *Dlk1* beyond a large LINE1-rich region), are preferentially expressed from the paternal allele (Figure 1A) (Tierling et al., 2009). *Dlk1* encodes delta like non-canonical Notch ligand 1. Paternal loss of the gene leads to partial neonatal lethality, and those animals that survive display post-natal growth retardation, increased adiposity, and skeletal defects (Moon et al., 2002). More recently, mice lacking *Dlk1* have also been shown to be prone to anxiety-like behaviors (García-Gutiérrez et al., 2018). *Rtl1* is a Ty3-gypsy retrotransposon-derived neogene that has evolved a function in placentation in eutherians. Loss of the paternal copy of *Rtl1* causes placental retardation and, in some mouse strains, can cause delayed parturition (Youngson et al., 2005; Sekita et al., 2008; Ito et al., 2015; Kitazawa et al., 2020; Kitazawa et al., 2021). The most distal imprinted gene in the domain, *Dio3*, encodes type 3 iodothyronine deiodinase, which is a negative regulator of thyroid hormone metabolism (Tsai et al., 2002). *Dio3* null mice show partial neonatal lethality and postnatal growth restriction. However, paternal loss of the gene leads to a much milder phenotype, reflecting the less stringent imprinting of this gene (Elena Martinez et al., 2014).

The maternally inherited chromosome expresses multiple imprinted noncoding transcripts, including *Gtl2* (also known as *Meg3*) (Figure 1). *Gtl2* is a long non-coding RNA (lncRNA) that is downregulated or lost in numerous human cancers, including breast and colorectal cancers (Makoukji et al., 2016; Buccarelli et al., 2020). *Gtl2* is also thought to form a long polycistronic transcript along with its associated transcripts *Rian* and *Mirg*, and acts as a host for multiple snoRNAs and miRNAs, including the miR-379/miR-410 cluster, all of which are driven by the *Gtl2* promoter (Cavaillé et al., 2002; Seitz et al., 2004; Tierling et al., 2006). In humans, the miRNAs in this cluster have been shown to be downregulated in the pancreatic islets of donors with type 2 diabetes (Kameswaran et al., 2014). Neonatal mice with a maternal deletion of the entire miR-379/miR-410 cluster are hypoglycemic and show impaired transition from fetal to postnatal metabolism (Labialle et al., 2014). These mice have also been shown to have increased anxiety-related behaviors in adulthood (Marty et al., 2016). Therefore, appropriate expression of genes in this region is essential for the lifelong health of mammals, and understanding the epigenetic regulation of these genes has significant biomedical relevance for a diverse range of processes.

**FIGURE 2:**
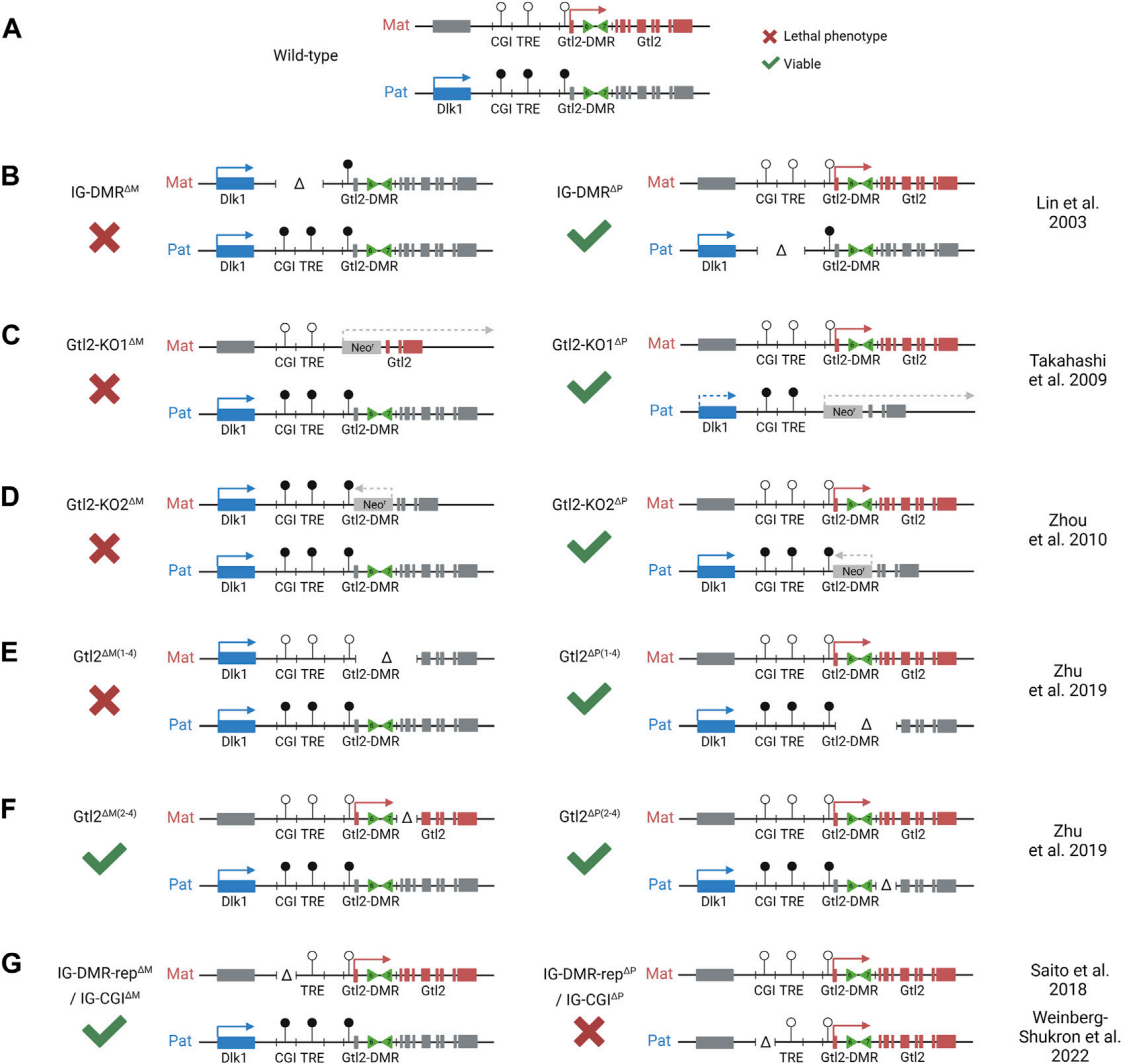
Integrated model depicting effects of different mice models with deletions at the Dlk1-Dio3 locus. Colored boxes represent expression from maternal (red) and paternal (blue) alleles. Gray boxes represent allelically repressed genes. Lollipops represent methylated (black) and unmethylated (white) regulatory elements. **(A)**. WT pattern of expression at the Dlk1-Dio3 locus. **(B)**. Maternal deletion of the entire IG-DMR results in a maternal-to-paternal epigenotype switch. **(C)**. Maternal replacement of *Gtl2* exons 1–7 with a forward-facing neomycin resistant cassette results in partial loss of the maternal gene expression. The same paternal substitution results in partial loss of the paternal gene expression. **(D)**. Maternal replacement of *Gtl2* exons 1–6 with a reverse facing neomycin resistant cassette results in loss of the maternal gene expression and activation of the maternal *Dlk1* expression. The same paternal substitution had no effect on the expression of methylation in the region. **(E)**. Maternal deletion of *Gtl2* exons 1–4 (including part of the Gtl2-DMR) results in a maternal-to-paternal epigenotype switch, which is similar to the IG-DMR deletion. However, methylation at the IG-DMR is not affected by this deletion. Paternal deletion has no effect. **(F)**. Neither maternal nor paternal deletion of *Gtl2* exons 2–4 has an effect on the expression of methylation at the Dlk1-Dio3 locus, indicating that the Gtl2-DMR, not the *Gtl2* gene, regulated imprinting at this region. **(G)**. An isolated paternal deletion of the IG-CGI results in a paternal-to-maternal epigenotype switch. Although maternal deletion has no effect, indicating that the IG-CGI is the primary methylation mark on the paternal chromosome, it is dispensable from the maternal chromosome.

**FIGURE 3:**
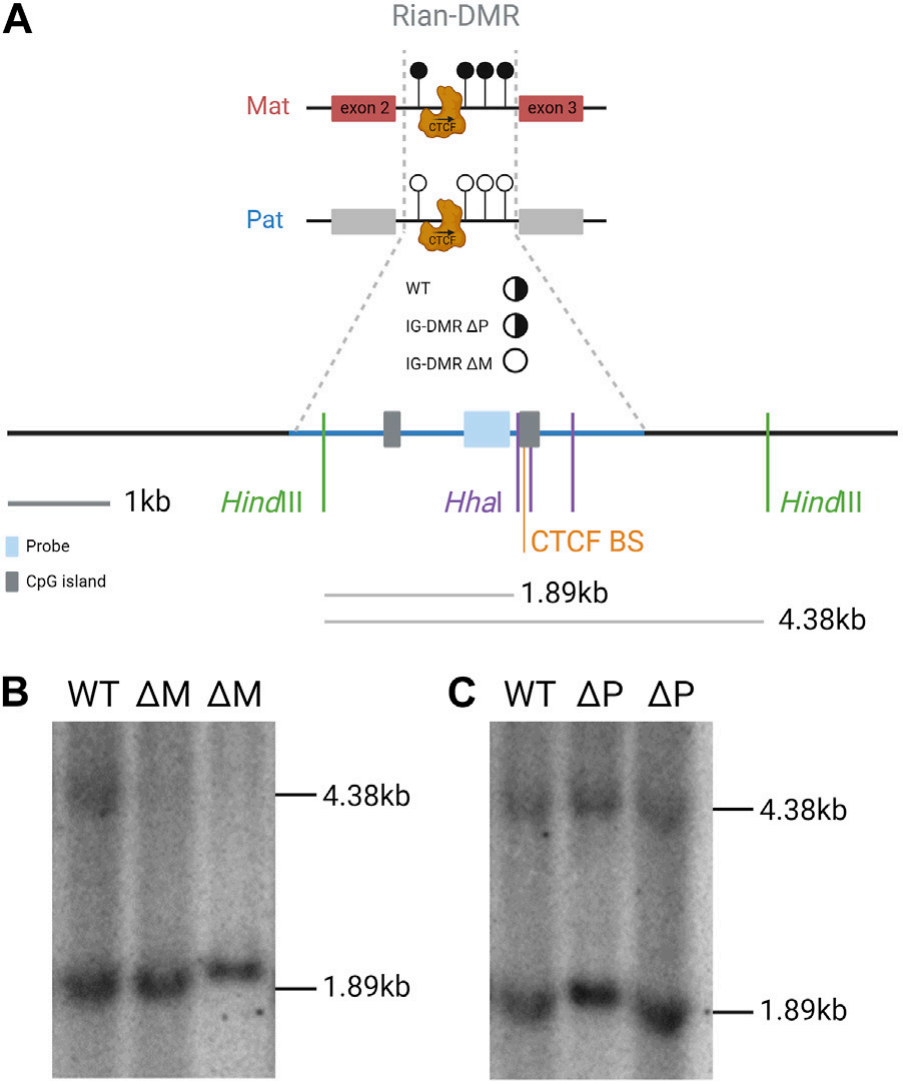
Rian-DMR is biallelically unmethylated in e16 embryos that inherit the IG-DMR deletion from their mother. **(A)**. Scale summary of the Southern blot displayed in part b relative to sequence features of the region and the location of the hybridization probe and the digest fragments that are hybridized with the probe. Full black circle, fully methylated; half black half white circle, differentially methylated; white circle, unmethylated. Blue shade, probe; gray shade, CpG islands; blue line, Rian-DMR +/-1 kb. **(B,C)**. Methylation-sensitive restriction-digested Southern blot of genomic DNA from e16 embryos hybridized with a Rian-DMR-specific probe. The genomic DNA was digested with *Hind*III in combination with *Hha*I in all lanes. WT, wild-type embryo; ΔM, IG-DMR maternally transmitted knockout embryo; ΔP, IG-DMR paternally transmitted knockout embryo.

Imprinting at the *Dlk1-Dio3* locus is regulated by the germline-derived intergenic DMR (IG-DMR) (Lin et al., 2003), which is required for regulating parent-specific expression in this locus (Rocha et al., 2008). This DMR is normally methylated in sperm and not methylated in oocytes, and maintains this parent-specific pattern throughout development (Figure 2A). After implantation, secondary somatic DMRs are established in the region: one at the promoter of the *Gtl2* gene (the Gtl2-DMR) and another tissue-specific partial DMR over the fifth exon of *Dlk1.* Both of these somatic DMRs are also hypermethylated on the paternal chromosome (Takada et al., 2000). A third somatic DMR is located in the second intron of *Rian* (known as *MEG8* in humans) (Zeng et al., 2014). This DMR is dependent on the IG-DMR, but in contrast to the other DMRs in the region, it gains secondary methylation on the maternally inherited chromosome (Figure 3). Here, we review the current knowledge on how differential epigenetic landscapes, genetic elements, and transcription are exquisitely coordinated to regulate genome function in this domain.

## Regulation and hierarchy of imprinting at the Dlk1-Dio3 region

The IG-DMR spans approximately 5 kb between *Dlk1* and *Gtl2*. Maternal deletion of this ICR region leads to paternalization of the maternal chromosome, and mice die between e16.5 and birth (Lin et al., 2003; Lin et al., 2007). However, on paternal transmission, the deletion has no effect (Figure 2B). The IG-DMR contains a small CpG island comprising seven tandemly repeated sequences, five of which contain a ZFP57 binding motif (Takada et al., 2002). The sequence-specific zinc finger protein, ZFP57, only binds to the methylated paternal chromosome, where it interacts with TRIM28, which in turn recruits the repressive epigenetic machinery, including DNMTs and heterochromatin-associated proteins such as SETDB1 and HP1, thereby maintaining the methylation memory of the germline imprint in early development in an environment where most epigenetic modifications elsewhere are being erased (Quenneville et al., 2011; Messerschmidt et al., 2012).

Recently, the IG-DMR was shown to be a bipartite element comprising two distinct functional elements (Aronson et al., 2021). In addition to the CpG island (IG-CGI) described earlier, it also contains a transcriptional regulation element (IG-TRE) that can bind pluripotency transcription factors in mouse embryonic stem cells (mESCs) and exhibit active enhancer marks (H3K27ac) and nascent transcription (Danko et al., 2015; Luo et al., 2016) (Figure 1C). It was, therefore, suggested that the IG-TRE serves as a putative enhancer, driving the expression of the maternally inherited genes within the domain.

Furthermore, these data also indicate that the IG-CGI is required to inactivate the paternal IG-TRE and maintain a repressive chromatin landscape on the paternal chromosome (Stelzer et al., 2016; Kojima et al., 2022; Weinberg-Shukron et al., 2022). Surprisingly, in contrast to the full IG-DMR deletion (maternal to paternal epigenotype switch but no effect upon paternal transmission), an isolated paternally derived deletion of the IG-CGI results in the reciprocal paternal-to-maternal epigenotype switch, with the IG-TRE becoming hypomethylated on the paternal chromosome (Saito et al., 2018) (Figure 2G).

Together, these findings indicate that the key element regulating imprinted expression on the maternal chromosome is the IG-TRE, which promotes activity from the maternally inherited non-coding RNAs, with the unmethylated IG-CGI being irrelevant for that function. In addition, the key element on the paternally inherited chromosome is a germline-methylated IG-CGI that is required for methylation and repression of the IG-TRE.

Some ICRs, including the IG-DMR, have also been shown to bind AFF3, a component of the super elongation complex-like 3 (SEC-L3), on the methylated allele in ESCs where it is thought to interact with the ZFP57/TRIM28 complex (Luo et al., 2016). However, the function of this interaction is not clear as depletion of AFF3 in ESCs leads to decreased expression of the maternally expressed genes in the *Dlk1-Dio3* region, demonstrating that it does not cooperate in protecting the IG-DMR from demethylation. Intriguingly, AFF3 also binds to a second region at the 3’ side of the IG-DMR downstream of the IG-TRE. Here, AFF3 is co-bound with ZFP281 but only on the unmethylated maternal copy (Wang et al., 2017). ZFP281 is a zinc finger protein that has previously been reported to act as both a transcriptional activator and a repressor (Wang et al., 2008). In the *Dlk1-Dio3* locus, depletion of ZFP281 from ESCs leads to decreased AFF3 binding at this downstream region, but not at the methylated IG-CGI. As depletion of AFF3 leads to decreased expression of *Gtl2*, *Mirg*, and *Rian*, this suggests that the downstream bound region is relevant for AFF3 function and that it also acts as an enhancer for maternally expressed genes (Wang et al., 2017). Whether this second region is acting in concert with the IG-TRE to control the expression of *Gtl2* and its associated transcripts remains to be established.

The combined results from the IG-DMR and the IG-CGI deletion indicate that the paternal IG-CGI is required to inactivate the paternal IG-TRE and maintain a repressive chromatin landscape on the paternal chromosome; this same element is dispensable on the maternal chromosome that is normally not methylated. On the other hand, the IG-TRE is dominant over the IG-CGI on the maternal chromosome where it is required to establish maternal gene expression and prevent methylation at the Gtl2-DMR. Together, the paradoxical effects imposed by distinct deletions within the IG-DMR represent an attractive experimental framework for dissecting the impact of changes in gene dosage on embryonic phenotypes. Synthesizing the result of the two genetic models shows that normal development cannot occur with biallelic expression of maternal genes and repression of *Dlk1* or with biallelic expression of *Dlk1* and repression of maternal transcripts. In both models, as in WT, monoallelic expression of the genes in this locus is consistent with normal development.

In accordance with that, flipping imprinting on both alleles produced viable offspring, showing that the parental origin of the imprint is irrelevant, provided appropriate balanced gene expression is established and maintained at this locus (Weinberg-Shukron et al., 2022). This has been demonstrated for another imprinted gene as well, where *Zdbf2* dosage, regardless of parental origin, regulates postnatal body weight (Glaser et al., 2022). These studies emphasize the importance of exquisite dosage control by genomic imprinting and the adaptability of this epigenetically regulated mechanism in particular developmental contexts (Liao et al., 2021).

## The role of the Gtl2-DMR and Gtl2 lncRNA in regional control

Monoallelic expression of *Gtl2/Meg3* exclusively from the maternally inherited chromosome is first observed in e3.5 blastocysts (Nowak et al., 2011; Sato et al., 2011). This imprinted expression precedes the acquisition of methylation at the *Gtl2* promoter on the paternal chromosome that is not observed until after e5.5. This suggests that transcription from the maternal promoter protects the maternal allele from gaining methylation and that methylation on the paternal chromosome occurs secondary to and in the absence of transcription. Once established, the Gtl2-DMR extends from the promoter into the first intron of the gene. Mouse models deleting the maternal Gtl2-DMR recapitulate the full ICR deletion, with the downregulation of maternally expressed genes, upregulation of paternally expressed genes, and embryos dying *in utero* (Takahashi et al., 2009; Zhou et al., 2010; Zhu et al., 2019) (Figures 2C–E). Furthermore, a patient with a maternal microdeletion of the Gtl2-DMR presented with features similar to UPD(14)Pat or maternal IG-DMR deletion patients (Kagami et al., 2010). Together, these data indicate that the unmethylated Gtl2-DMR on the maternal chromosome, once established, is able to act as an imprinting control region for the entire domain, but it is unclear whether this is by the Gtl2 lncRNA itself or *via* direct *cis*-acting elements within the DMR.

The *Gtl2* gene transcribes a long non-coding RNA whose function as a regulatory transcript continues to be explored, and several different roles have been proposed. *MEG3*, the human ortholog of *Gtl2*, is downregulated in many forms of cancer and, therefore, is believed to function as a tumor suppressor (Makoukji et al., 2016; Buccarelli et al., 2020). The *MEG3* lncRNA has been shown to interact with another tumor suppressor, p53, and influence the expression of p53 target genes (Zhou et al., 2007; Zhu et al., 2015). The lncRNA has also been found to interact with the polycomb repressive complex 2 (PRC2) in both mouse and human cells (Zhao et al., 2010; Mondal et al., 2015). In humans, *MEG3* exon3 is thought to contain the region of interaction with PRC2; this exon is conserved with mouse exon3, suggesting a shared function between the two species. *MEG3* is then thought to recruit PRC2 to its target genes in *trans* though interaction with GA-rich repeat regions and formation of RNA–DNA triplexes (Mondal et al., 2015).

**FIGURE 4:**
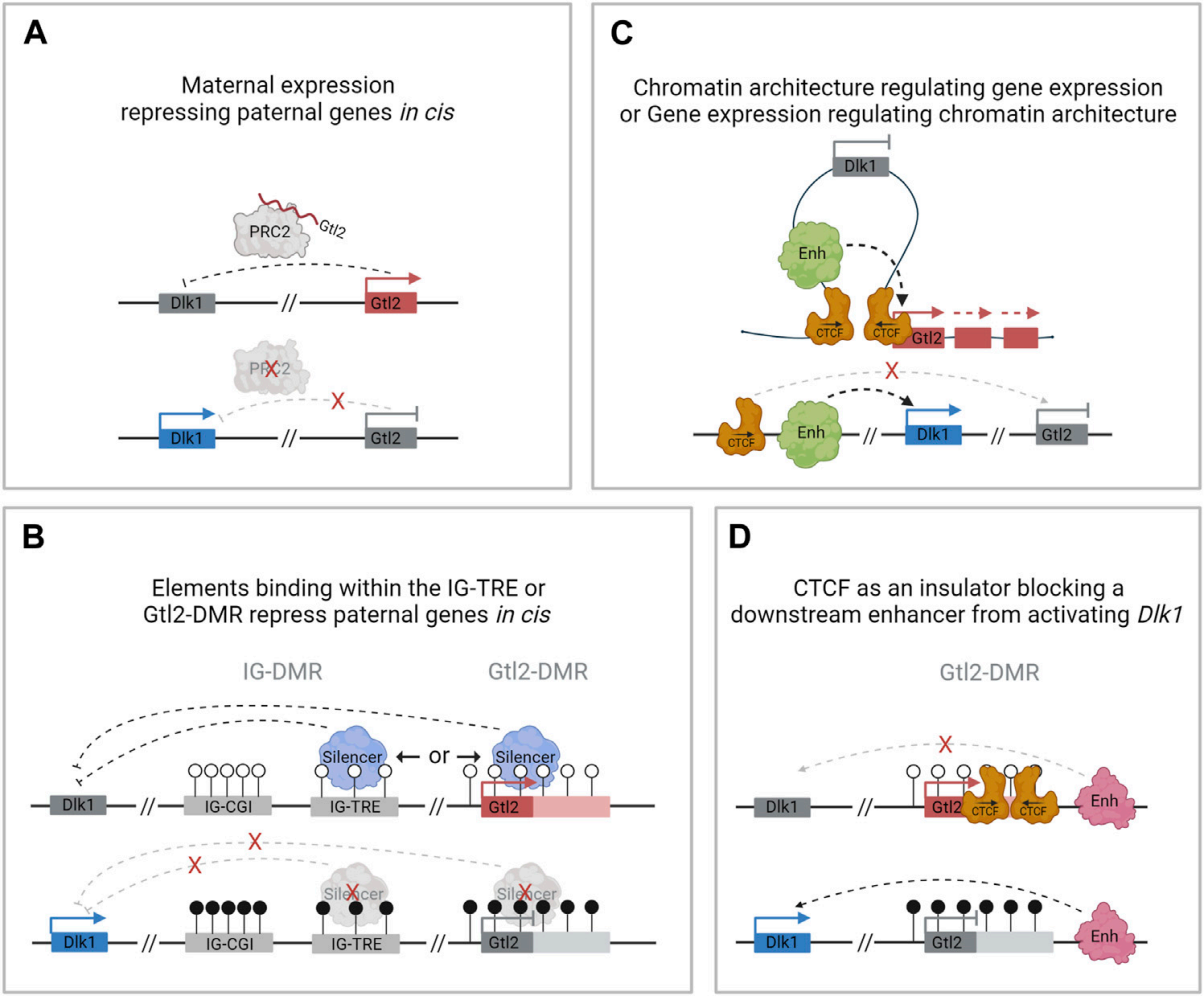
Possible mechanisms of regulation at the Dlk1-Dio3 locus by *Gtl2.* **(A)**. *Gtl2* lncRNA facilitates PRC2 recruitment in *cis* and represses paternally expressed genes on the maternally inherited chromosome. **(B)**. The IG-TRE or the Gtl2-DMR harbors elements that directly repress paternal genes in *cis*. **(C)**. Differential CTCF binding at the Gtl2-DMR regulates gene expression by restricting access to shared enhancers. **(D)**. Differentially methylated CTCF binding sites at the Gtl2-DMR function as an insulator, preventing *Dlk1* from being expressed from the maternal chromosome.

In other imprinted regions, lncRNAs have been shown to silence other genes in the domain in *cis* (Pauler et al., 2012), and in the Dlk1-Dio3 domain, *in vivo* manipulations that activate *Gtl2* on the paternal chromosome result in repression of *Dlk1* (Lin et al., 2003; Li et al., 2008; Weinberg-Shukron et al., 2022). Furthermore, knockdown of the *Gtl2* lncRNA leads to increased expression of *Dlk1* coupled with decreased histone H3K27me3 over the *Dlk1* gene in mouse ESCs (Zhao et al., 2010). This suggests that in mice, the *Gtl2* lncRNA may facilitate PRC2 recruitment *in cis* and that one of its major functions is to repress paternally expressed genes on the maternally inherited chromosome (Figure 4A). However, in human iPSCs lacking *MEG3* expression, no difference was observed in PRC2 occupancy over the *DLK1* promoter, and there were no significant changes in the expression levels of either *DLK1* or *DIO3* compared with iPSCs that express *MEG3* (Kaneko et al., 2014). It should be noted that these experiments were performed *in vitro* in cell lines where *Dlk1* is only weakly expressed.

More recently, mouse models have also caused doubt regarding the idea that the *Gtl2* lncRNA silences the *Dlk1-Dio3* domain *in vivo*. Whereas a deletion model that removes *Gtl2* exons 1–4 leads to the loss of imprinting in the whole domain (Figure 2E), deleting exons 2–4 causes downregulation of *Gtl2* but no change in *Dlk1* expression in e11.5 embryos (Figure 2F) (Zhu et al., 2019). This suggests that the Gtl2-DMR does not solely function to restrict *Gtl2* expression to the maternal chromosome and that the lncRNA does not silence the paternally expressed genes on the maternal chromosome *in cis*. Instead, these observations suggest that the Gtl2-DMR harbors elements that can directly repress the expression of the paternally expressed genes *in cis* (Figure 4B). In agreement with this hypothesis are data from ESC deletions. Sanli et al. (2018) made ESC lines lacking either the *Gtl2* promoter or intron 1. Intriguingly, the loss of intron 1 alone on the maternal chromosome was sufficient to silence *Gtl2* and all the associated maternally expressed non-coding transcripts in ESCs and upon differentiation to NPCs. Furthermore, *Dlk1* expression became biallelic in NPCs upon the loss of the maternal intron 1, indicating that *Gtl2* intron 1 may be playing a vital role in imprinting control across this domain.

*Gtl2* intron 1 is approximately 2.5 kb in length. It contains two CTCF binding sites that are conserved in eutherian mammals and are only able to bind CTCF on the unmethylated maternally inherited chromosome (Lin et al., 2011) (Figure 1D). CTCF plays an important role in genome organization and is frequently found at boundaries of topologically associating domains (TADs) which are self-interacting regions (Merkenschlager and Nora, 2016). Although TADs are thought to be maintained between different tissues, sub-TADs within them are tissue-specific and orchestrate local genomic contacts throughout development (Smith et al., 2016). Data from ESCs indicate that the *Gtl2* CTCFs form the boundary of a parent-of-origin-specific sub-TAD on the maternal chromosome (Llères et al., 2019). Thus, another possible mechanism for establishing differential expression profiles between the two parental chromosomes could be that differential CTCF binding contributes to the formation of parental-origin-specific regulatory conformations (Figure 4C). Another possibility is that access to shared enhancers might be insulated through CTCF binding, enabling *Gtl2* expression and *Dlk1* repression (Figure 4D) consistent with a similar mechanism that is well established for the *Igf2*/*H19* domain, where imprinted gene expression is controlled by differentially methylated CTCF binding sites in the ICR. The H19-CTCF sites are methylated on the paternally inherited chromosome and are thus only able to bind CTCF on the maternal chromosome, where they function as an insulator, preventing *Igf2* from being expressed (Bell and Felsenfeld, 2000; Hark et al., 2000). In ESCs, the H19-ICR has been recently shown to form a maternal chromosome-specific sub-TAD boundary that splits the imprinted domain into two, which is similar to what is observed in the *Dlk1*-*Dio3* locus. Deletion of one of the CTCFs *in vitro* has been shown to cause upregulation of *Dlk1* (Llères et al., 2019). However, the extent to which the *Gtl2* CTCFs can regulate gene expression *in vivo* remains to be established.

## The Rian-DMR: A paternal chromosome-specific regulatory element?

*Rian* (RNA imprinted and accumulated in the nucleus) is a lncRNA that has more than 20 predicted alternative transcripts in mice. Its expression from the maternal chromosome has not been dissociated from *Gtl2*, and an individual promoter for this gene has not been identified or a transcription unit clearly defined; hence, it can be considered as a *Gtl2*-associated transcript driven by the IG-DMR and Gtl2-DMR. Nonetheless, RNA from this region acts as the host transcript for miRNAs and two clusters of C/D snoRNAs (Cavaillé et al., 2002). In humans, the *Rian* ortholog, *MEG8* (along with *MEG3*), has been shown to be upregulated in many cancers and is thought to regulate many different pathways by acting as a molecular sponge for various miRNAs (Ghafouri-Fard et al., 2022). A Rian-DMR has been described in the second intron of the gene (Figure 1E; Figure 3A), and as opposed to other DMRs, in the *Dlk1-Dio3* domain, this region is methylated in both sperm and oocytes, and then becomes hypomethylated in the blastocyst. The paternally inherited allele remains hypomethylated throughout development, whereas the maternally inherited copy becomes hypermethylated by e6.5 (Zeng et al., 2014). This hypermethylation on the maternal allele may be due to the normal methylation accumulation on actively transcribed gene bodies. Upon maternal transmission of the IG-DMR deletion, the Rian-DMR is lost; however, unlike the Gtl2-DMR, the Rian-DMR becomes biallelically hypomethylated (Figure 3B). This once again illustrates that appropriate methylation of the germline ICR is necessary to establish the epigenotype of the entire domain.

In mice, the DMR consists of a small CpG island that contains 12 copies of a GGCG repeat. This region is conserved and G-rich in eutherian mammals; however, the GGCG repeat is only seen in mice and rats. Upstream of the repeats is a conserved CTCF binding domain. Interestingly, this motif lacks CpG dinucleotides, so binding is not affected by methylation. In agreement with this, CTCF occupancy has been shown to be biallelic at this site (Zeng et al., 2014). Until now, the role of the *Rian*-DMR has been unclear, but a recent study has thrown some light on its function. Han et al. (2022) have shown that the DMR functions as an insulator in mouse MLTC-1 cells. Intriguingly, the CTCF binding region was only able to act as an insulator in the presence of the repeat element. They further deleted the entire DMR, and the CTCF site repeats individually to assess the role of the region on gene expression. A 661bp deletion of the entire DMR led to reduced *Dlk1* and *Rtl1* expression and increased expression of *Gtl2*, *Rian*, and *Mirg*. When the CTCF binding site alone was deleted, a similar but less pronounced effect was observed. Interestingly, the tandem repeat deletion only affected the expression of the downstream gene *Mirg.* These data indicate that the Rian-DMR functions on the unmethylated paternal chromosome to ensure the correct expression of *Dlk1* and *Rtl1* and the repression of *Gtl2* and its associated transcripts (Zeng et al., 2014). However, more recent *in vivo* data from mice with a 434bp deletion of the CTCF binding site and the GGCG repeats show that the loss of this region has little phenotypic effect as maternal and paternal heterozygotes and homozygotes all survive to adulthood. Furthermore, no effect was observed at e12.5 on *Dlk1*, *Rtl1*, *Dio3*, *Gtl2*, *Rian*, or *Mirg* expression on either maternal or paternal transmission of the deletion. These mice do show increased expression of two miRNAs within the *Rian* gene, miR-118 and miR-341. Both miRNAs are significantly upregulated in paternal heterozygotes and homozygotes but not in maternal heterozygotes at e12.5. In addition, RNAseq data indicated that many other miRNAs in the region become upregulated on paternal deletion, suggesting a role of the Rian-DMR in preventing miRNA expression on the paternal chromosome (Zhang et al., 2023).

## The Dlk1-DMR and *Dlk1* isoforms

Both the promoter of *Dlk1* and the last exon of *Dlk1* contain CpG islands (Takada et al., 2000). Whereas the *Dlk1* promoter does not show any parental-origin-specific methylation pattern, the smaller CpG island within the fifth exon is completely unmethylated on the maternal allele and partially methylated on the paternal allele (Takada et al., 2002). This differentially methylated region is termed the Dlk1-DMR (Figure 1B). Similar to the Gtl2-DMR, the Dlk1-DMR acquires paternal allele-specific methylation following fertilization (Gagne et al., 2014). The methylation pattern of this DMR remains dynamic in late embryonic development and into adulthood. Interestingly, the level of Dlk1-DMR methylation does not correlate with the level of *Dlk1* expression. Takada et al. (2002) reported a different methylation profile per tissue (lung, muscle, liver, kidney, and brain), suggesting that the methylation is cell-type specific and that allele-specific methylation differences at the Dlk1-DMR may not have a role to play in transcriptional control. The function of the Dlk1-DMR remains to be elucidated.

Alternative splicing at exon 5 generates a membrane-bound and secreted isoform of *Dlk1*. The secreted isoforms, produced with a longer part of exon 5, include a juxtamembrane motif for cleavage by extracellular proteases, which is absent from constitutively membrane-bound isoforms. In the neurogenic niche, secreted *Dlk1* is predominantly expressed by niche astrocytes, whereas neural stem cells (NSCs) express membrane-bound *Dlk1* (Ferrón et al., 2011). Interestingly, membrane-bound DLK1 in NSCs is stimulated by astrocyte-secreted DLK1, and communication between these cell types in the neurogenic niche regulates NSC self-renewal.

## Selective absence of imprinting of *Dlk1* not *Gtl2* in the neurogenic niche

Recent evidence suggests that imprinted genes can be selectively “switched on” or “switched off” in particular cell types or at specific developmental time-points to initiate a change in gene dosage that is essential for normal development (Ferrón et al., 2011; Ferrón et al., 2015). Intriguingly, some imprinted genes show a selective absence of imprinting in the neurogenic niche (Lozano-Ureña et al., 2017). The *Igf2* gene, which is canonically expressed from the paternally inherited copy, is biallelically expressed in the choroid plexus (DeChiara et al., 1991; Giannoukakis et al., 1993; Lehtinen et al., 2011), and this selective absence of *Igf2* imprinting is required for neurogenesis.

**FIGURE 5:**
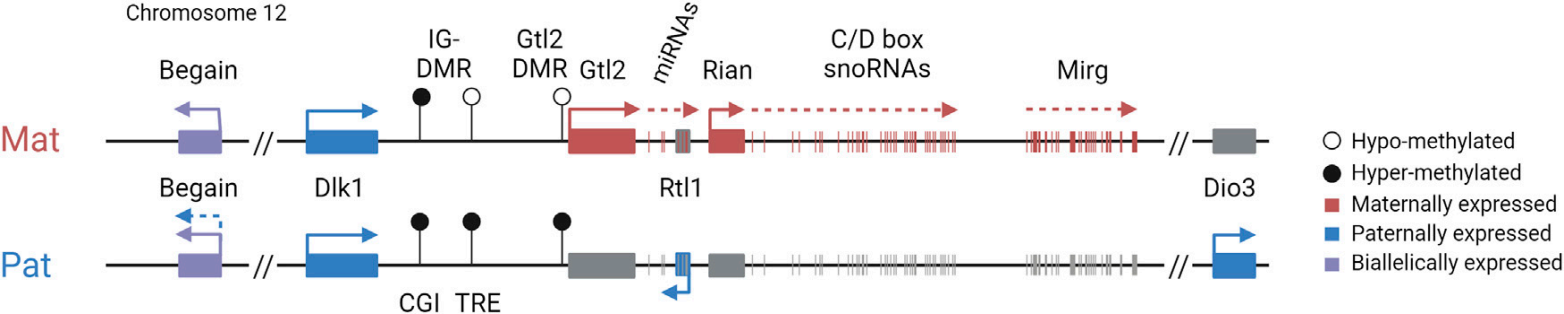
Selective absence of imprinting in the neurogenic niche; in the postnatal subventricular zone, niche astrocytes and neuronal stem cells exhibit biallelic expression of *Dlk1*, whereas *Gtl2* retains imprinting and remains exclusively maternally expressed. This selective absence of imprinting is accompanied by increased methylation at the IG-CGI but not at the IG-TRE or Gtl2-DMR.

The vertebrate-specific atypical Notch ligand gene, *Dlk1*, is dosage-sensitive with different tissue-specific sensitivities to altered expression levels (Moon et al., 2002; Da Rocha et al., 2009). DLK1 is involved in a range of processes, including non-shivering thermogenesis, metabolism, and behavior (Wallace et al., 2010; Charalambous et al., 2012; García-Gutiérrez et al., 2018; Montalbán-Loro et al., 2021). In humans, *DLK1* variants are associated with age at menarche (Day et al., 2017), type I diabetes (Wallace et al., 2010), and a range of cancers, including neural, breast, and liver cancer (Yin et al., 2006; Cai et al., 2016; Makoukji et al., 2016; Buccarelli et al., 2020). DLK1 is, therefore, a biomedically relevant key player in a diverse range of processes. Similar to *Igf2*, the *Dlk1* gene shows selective absence of imprinting in the postnatal neurogenic niche, resulting in the activation of the repressed maternal allele *via* an unknown mechanism (Figure 5). This absence of imprinting is essential for normal adult neurogenesis (Ferrón et al., 2011). Unlike *Dlk1,* the neighboring gene *Gtl2* keeps its imprinting in the neurogenic niche, suggesting a selective gene-specific regulation (Ferrón et al., 2011).

An important evolutionary question remaining to be elucidated is the time of switch in imprinted gene dosage. Although the dosage change is described as a selective absence of imprinting, it is not known whether biallelic expression of *Dlk1* is “switched on” in specific cell types, representing a recently evolved function, or whether imprinting is “switched off” during development, representing an ancestral state prior to the evolution of imprinting, where *Dlk1* is biallelically expressed (Edwards et al., 2008). Remarkably, this selective requirement for a double dose for neurogenesis is shared by the imprinted *Igf2* gene (Ferrón et al., 2015). This emphasizes the importance of exquisite dosage control of certain genes by genomic imprinting, and the adaptability and flexibility of this epigenetically regulated mechanism in particular developmental contexts.

In conclusion, selective regulation of imprinting is probably a normal mechanism for modulating gene dosage to control stem cell potential in brain development and within the neurogenic niches throughout development and adult life (Perez et al., 2016). The dosage sensitivity of functionally important imprinted genes and the finding of highly selective absence of imprinting at *Dlk1* and *Igf2* in the brain suggest tight regulation of parental-origin-specific monoallelic expression. Dissecting the molecular players that participate in regulating imprints during postnatal neurogenesis will provide insights into the wider epigenetic control of the neurogenic process and uncover the molecular mechanisms underlying normal NSC function to understand tumoral processes in the adult brain. Therefore, unmasking the mechanism that regulates this time- and tissue-specific change in gene dosage is crucial for expanding our understanding of the physiological pathways regulated by imprinted genes in pathology and health.

## Discussion

### How to build an imprinted domain

Clearly, appropriate allele- and tissue-specific expression of the Dlk1-Dio3 region is necessary for normal mammalian development. Assessing different models that remove various elements in the region has allowed us to dissect the chain of events that is necessary to establish and maintain imprinted gene expression.

**FIGURE 6:**
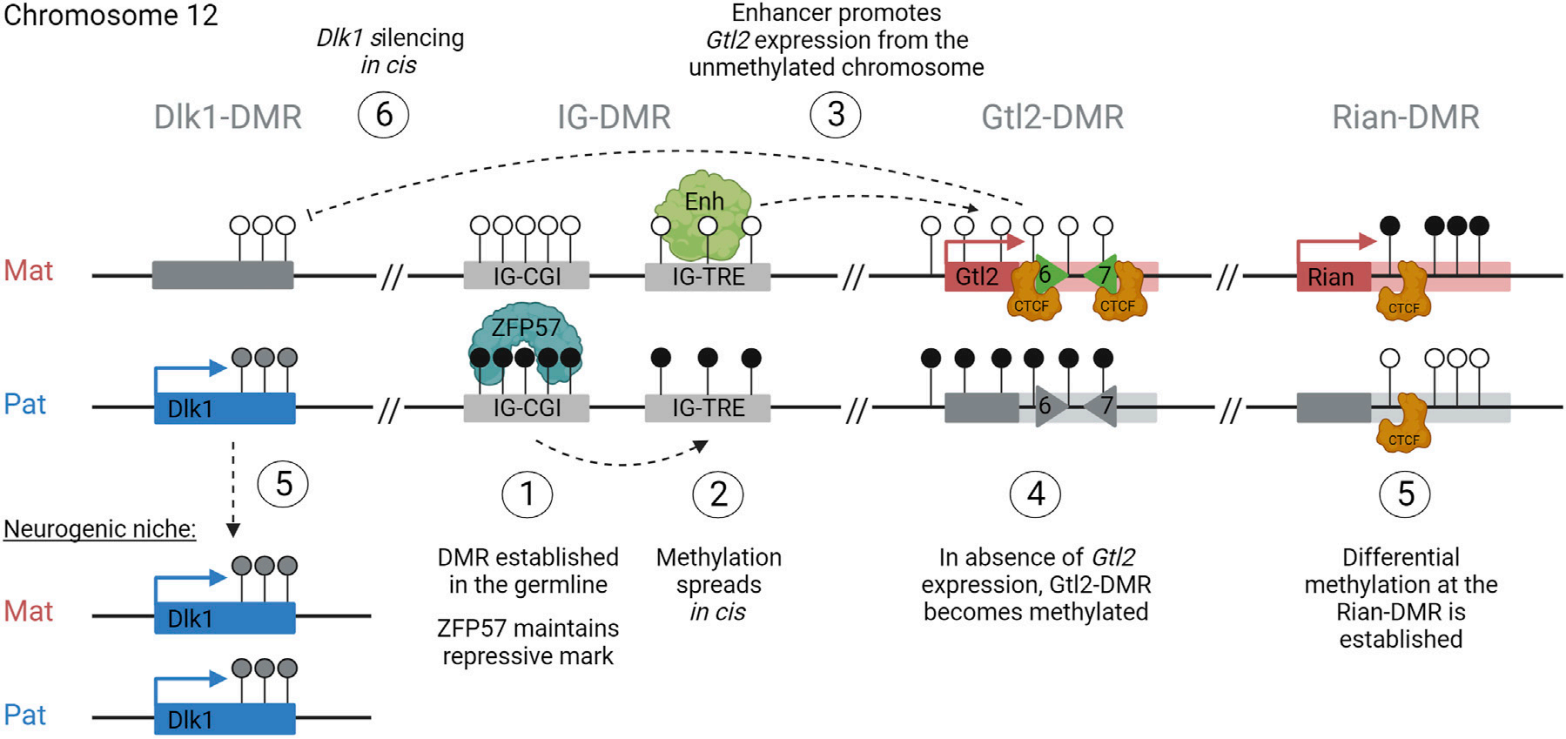
How to build an imprint: step 1: establishment of the germline-DMR (IG-CGI), methylated in sperm and unmethylated in oocytes. Step 2: maintenance of methylation across the entire paternal ICR (CGI + TRE). Step 3: monoallelic expression of *Gtl2* from the maternal unmethylated allele. Step 4: accumulation of methylation over the paternal Gtl2-DMR. Step 5: establishment of somatic DMRs at *Dlk1* and *Rian*. Step 6: regulation of gene expression profiles in *cis*, silencing *Dlk1* from the maternal copy.

The first stage of the hierarchy is the establishment of the germline-DMR. In sperm, the IG-DMR becomes fully methylated before e19.5 (Hiura et al., 2007), whereas in oocytes, the region remains unmethylated (Figure 6, step 1). After fertilization, the presence of DNA methylation across the paternal IG-CGI allows the recruitment of oocyte-loaded and zygotically expressed ZFP57 to the region, which in turn recruits TRIM28 and DNMTs and ensures that the entire ICR, including the IG-TRE, remains unmethylated on the paternal chromosome (Figure 6, step 2). Meanwhile, on the maternally inherited chromosome, the unmethylated IG-CGI is unable to bind ZFP57 and the IG-TRE remains unmethylated. This allows the IG-TRE to bind transcription factors and act as an enhancer for *Gtl2*, causing it to be monoallelically expressed from the maternally inherited chromosome from e3.5 (Figure 6, step 3). DNA methylation starts to accumulate over the paternal copy of the Gtl2-DMR at e5.5 and is complete by e6.5 (Figure 6, step 4). At the same time, the other somatic DMRs are also established at *Dlk1* and *Rian* (Figure 6, step 5). Once established, the Gtl2-DMR can control imprinted gene expression on the maternal chromosome either *via* the lncRNA recruiting PRC2, direct silencing, or insulator activity (Figure 6, step 6). On the paternal chromosome, the unmethylated Rian-DMR regulates paternal miRNA expression, possibly through its insulating properties.

### Remaining questions

Although much has been learned about how imprinting is established in this domain, many questions remain. First, it is not clear how the IG-DMR becomes methylated in the male germline yet remains unmethylated in oogenesis and how the maternal copy eludes *de novo* methylation in later development. Recently, it was shown that a mouse IG-DMR transgene acquired methylation during the post-fertilization period rather than in the sperm (Matsuzaki et al., 2023). This suggests that the transgene is lacking the sequence that initially attracts methylation to the element in the sperm. This “post-fertilization imprinted methylation” was previously reported in mouse and human *H19* ICR transgenes as well (Matsuzaki et al., 2009). However, after implantation, the YAC transgene of the IG-DMR became highly methylated from both copies, suggesting that the IG-DMR fragment tested did not protect the maternal IG-DMR from genome-wide *de novo* DNA methylation. Interestingly, the fragment did not contain most of the IG-TRE, indicating that one function of the IG-TRE may be to protect the maternal sequence from global *de novo* methylation after implantation. Although maintenance of hypomethylation at the maternal *H19* ICR is known to involve CTCF and Sox/Oct factors (Sakaguchi et al., 2013), the mechanism at the maternal IG-DMR is not fully understood; however, this region also contains Sox/Oct binding motifs.

Second, the mechanism by which the maternal IG-TRE directs monoallelic expression in the domain remains to be elucidated. It is known to contain many transcription factor binding motifs and shows low-level expression, suggesting that it most likely functions as an enhancer for *Gtl2*. However, it is possible that the IG-TRE also contains a silencer element that is capable of directly repressing the paternally expressed genes on the maternally inherited chromosome. Experiments dissecting this region further are necessary to tease apart these options.

Evidence from mice harboring deletions of the Gtl2-DMR and *in vitro* deletions of the Rian-DMR indicate that somatic DMRs can regulate parent-of-origin-specific expression in later development, but the mechanisms through which this is achieved are not fully understood. Intriguingly, both these DMRs are known to bind CTCF, so they may influence gene expression through mechanisms such as enhancer blocking. The parental-specific sub-TAD identified *in vitro* with the Gtl2-DMR at the border indicates that it has strong insulator activity. However, whether these parental-specific conformations are a cause or a consequence of differential expression patterns between the two chromosomes is uncertain (Figure 6). The role the *Gtl2* lncRNA itself plays in the regulation of gene expression in the region also needs further exploration as it is uncertain whether it recruits PRC2 to the domain *in vivo* to bring about the epigenetic silencing of genes. Much of the research on conformation and the role of the lncRNA at the *Dlk1-Dio3* locus has been performed *in vitro*, limiting the resolution of information, and thus, findings may not be recapitulated *in vivo*. For instance, as *Dlk1* is lowly expressed in mESCs, experiments designed to look at the effect of perturbation models on paternal gene expression patterns are not informative in culture.

Finally, the Dlk1-Dio3 locus is interesting as the expression varies between tissues and cell types. We recently showed that there are two weakly biased genes at the edge of the *Dlk1-Dio3* region: *Wdr25* and *Wars*. Both genes showed a weak skew toward paternal expression, but only in brain tissues. This bias was shown to be under the control of the IG-DMR (Edwards et al., 2023), suggesting that its influence may be more extensive in neuronal tissues. Weakly biased genes were also found at the periphery of other imprinted regions, and further studies are needed to understand the functional and mechanistic implications of this observation. In addition to tissue-specific differences, unique cell types display selective absence of imprinting at the *Dlk1-Dio3* domain in a temporal and spatial-specific manner. The mechanism that switches between monoallelic and biallelic expression remains to be elucidated and may provide insights into transcriptional control with wider implications for non-imprinted domains as well (Figure 6). Together, these observations indicate that the mechanisms regulating the imprinting of the *Dlk1-Dio3* locus may vary between tissues and time-points in development.

This review highlights the importance of using *in vivo* models to tease apart the complex chain of epigenetic events that is required to establish and maintain imprinted gene expression throughout development. We also demonstrate that cell-type-specific modulation of this hierarchy is necessary to ensure the correct gene dosage in certain tissues, such as in the neurogenic niche—however, what these mechanistic steps are remains unclear. Together, this work illustrates how studying one imprinted region in detail can add a layer of sophistication to how we think about the epigenetic control of genome function and its consequences for spatial and temporal regulation more generally.

## Methods

*Southern blot* (*Figure 3*): DNA was isolated by standard techniques (Sambrook et al., 2001). A total of 10 μg of restriction enzyme-digested DNA was separated on a 0.5% TBE gel before transferring to Hybond-N+ (GE Healthcare Life Sciences) nylon membranes. Membranes were pre-hybridized in ULTRAhyb (Ambion) for at least 1 h. The probe was a PCR fragment amplified with 5′-AGT​GGC​CCA​ACT​TCT​ATC​GG and 5′-GGA​ACA​GAG​ACC​TCC​TAA​GG, which was labeled with [α-32P]dCTP using the Megaprime DNA labeling system (GE Healthcare Life Sciences) and then purified with ProbeQuant G50 Micro-Columns (GE Healthcare Life Sciences) before being added to the hybridization solution and incubated at 42°C overnight. Filters were washed to a stringency of 0.2X SSC/0.1%SDS at 65°C and then exposed to PhosphorImager Screens (Molecular Dynamics).
